# Exploring the Use of Thermal Infrared Imaging in Human Stress Research

**DOI:** 10.1371/journal.pone.0090782

**Published:** 2014-03-27

**Authors:** Veronika Engert, Arcangelo Merla, Joshua A. Grant, Daniela Cardone, Anita Tusche, Tania Singer

**Affiliations:** 1 Max Planck Institute for Human Cognitive and Brain Sciences, Department of Social Neuroscience, Leipzig, Germany; 2 Institute of Advanced Biomedical Technologies (ITAB), G. d'Annunzio Foundation, Chieti, Italy; 3 Department of Neuroscience and Imaging, G. d'Annunzio University, Chieti-Pescara, Italy; University of Texas School of Public Health, United States of America

## Abstract

High resolution thermal infrared imaging is a pioneering method giving indices of sympathetic activity via the contact-free recording of facial tissues (thermal imprints). Compared to established stress markers, the great advantage of this method is its non-invasiveness. The goal of our study was to pilot the use of thermal infrared imaging in the classical setting of human stress research. Thermal imprints were compared to established stress markers (heart rate, heart rate variability, finger temperature, alpha-amylase and cortisol) in 15 participants undergoing anticipation, stress and recovery phases of two laboratory stress tests, the Cold Pressor Test and the Trier Social Stress Test. The majority of the thermal imprints proved to be change-sensitive in both tests. While correlations between the thermal imprints and established stress markers were mostly non-significant, the thermal imprints (but not the established stress makers) did correlate with stress-induced mood changes. Multivariate pattern analysis revealed that in contrast to the established stress markers the thermal imprints could not disambiguate anticipation, stress and recovery phases of both tests. Overall, these results suggest that thermal infrared imaging is a valuable method for the estimation of sympathetic activity in the stress laboratory setting. The use of this non-invasive method may be particularly beneficial for covert recordings, in the study of special populations showing difficulties in complying with the standard instruments of data collection and in the domain of psychophysiological covariance research. Meanwhile, the established stress markers seem to be superior when it comes to the characterization of complex physiological states during the different phases of the stress cycle.

## Introduction

Everyone has experienced the “rush of blood to the head” when confronted with a stressful situation. This form of stress reaction, i.e., skin blood flow, can be measured with the pioneering method of high resolution thermal infrared (IR-) imaging. IR-imaging estimates variations in autonomic activity reflected by a complex interplay of heat exchange processes involving skin tissue, inner tissue, local vasculature and metabolic activity [1]–[5]. In detail, during threat or arousal the sympathetic nervous system causes sweat secretions that lubricate the skin, achieving elasticity [6], [7] and sustaining temperature homeostasis in prolonged periods of vigorous activity [8]–[11]. Furthermore, vasoconstriction of the skin's blood vessels protects the body from possible hemorrhage and excessive blood loss during injury [12]–[15]. These physiological occurrences cause skin temperature to fluctuate. Thus, by observing the thermal infrared signal one can infer autonomic arousal and further attempt to differentiate between the two competing subdivision of the autonomic nervous system.

Compared to established stress markers like heart rate or the hypothalamic-pituitary-adrenal (HPA) axis end-product cortisol, the great advantage of IR-imaging is its non-invasiveness. The contact-free recording of facial tissues with an easy-to-hide thermal camera helps avoid sources of unsystematic data variation (e.g., participants' knowledge of being tested or the stressful installation of recording equipment on the participants' body). This opens up exciting research opportunities in the study of special populations (i.e., showing difficulties in complying with the standard instruments of data collection). Despite a growing interest in the method, IR-imaging has yet to find access to the field of stress research. We here tested the use of IR-imaging in the stress laboratory setting. In detail, we examined the sensitivity of IR-imaging data (hereafter referred to as facial thermal imprints) to two widely used laboratory tests, the Cold Pressor Test (CPT) and the Trier Social Stress Test (TSST). The CPT [16] is a pain induction technique; the TSST [17] a psychosocial challenge. Both are considered stress tests and reliably induce sympathetic and HPA-axis activity (for CPT-related evidence see [18]–[20]; for TSST-related evidence see [21]–[23]. Rather than use a single stressor and focus on test-retest reliability we chose to cover a wider spectrum of stressors (psychosocial versus physical). This decision was driven by the fact that it is difficult to achieve robust stress responses when repeatedly administering a single stress test.

Fifteen healthy males underwent CPT and TSST in pseudo-randomized order. Women were excluded to avoid the confounding effects of hormonal status on cortisol levels [24]. Facial thermal imprints were collected across anticipation, stress and recovery phases of both stress tests. To allow for a comparison of thermal imprints with established stress markers, we further assessed the participants' heart rate (regulated by sympathetic and parasympathetic influences), heart rate variability (HRV; permitting to disentangle sympathetic and parasympathetic contributions to heart rate) and finger temperature (indicative of adrenergic sympathetic activity). The salivary enzyme alpha-amylase as an indirect indicator of adrenergic sympathetic activity and the hormone cortisol as an indicator of HPA-axis activity were additionally sampled from saliva (see Table 1 for a summary of the dependent variables). Conventional statistical tests and advanced multivariate pattern analyses (MVPAs) were applied to analyze the resulting set of dependent variables over time. The MVPAs aimed at exploring the relative sensitivity of thermal imprints and established stress markers for characterizing the different phases of the stress cycle.

**Table 1:** Summary of the dependent variables.

Variable	Description
**Facial thermal imprints**	
FHT	Forehead temperature
CRT	Corrugator temperature
POBT	Periorbital temperature
NTT	Nose tip temperature
POT	Perioral temperature
CHT	Chin temperature
**Established stress markers**	
HR	Heart rate
LF	Low frequency heart rate variability
HF	High frequency heart rate variability
LH/HF	Ratio of low to high frequency heart rate variability
FT	Finger temperature
AA	Alpha-amylase
COR	Cortisol

In summary, we could show that the majority of the facial thermal imprints were change-sensitive in CPT and TSST. Moreover, and in contrast to the established stress markers, the thermal imprints correlated with stress-induced mood changes. The established stress markers conversely had an improved ability to disambiguate anticipation, stress and recovery phases of both stress tests.

## Materials and Methods

### Participants

Male participants between 21 and 33 years of age were recruited by posting ads on an electronic billboard of Leipzig city. Women were excluded to avoid the confounding effects of hormonal status on cortisol levels [24]. Given a potential influence on our dependent variables, information about recreational drug use, medical and psychological history were assessed in a telephone interview. Regular recreational drug users (cannabis within the last two months, other recreational drugs within the past year), habitual smokers (>5 cigarettes/week) and individuals reporting chronic illness (e.g., cardiovascular or thyroid conditions) psychological disorders (e.g. depression or anxiety) or taking medication that influences HPA-axis regulation were excluded from the study. 15 participants (mean age 26.18 years; SD 3.36), all of which had college degrees and BMIs ranging between 21 and 25, were included in the study. The study was approved by the Research Ethics Board of Leipzig University (ethics number: 360-10-13122010) and performed in agreement with the Declaration of Helsinki. All participants signed informed consent and could withdraw from the study at any time.

### Cold Pressor Test

The CPT [16] is a widely used paradigm that has been applied to the study of pain since the 1930s [25]. Participants place their non-dominant hand or forearm into a tank of cold water (0–10°C) and are instructed to remove it when the sensation becomes intolerable. Typically, a ceiling of several minutes for hand/forearm submersion is imposed. For the current study, the main unit of the CPT device consisted of a “Mobicool” cooler (model B40) with a volume of 40 L which allowed for automatic temperature maintenance (at 3°C (+/−1°C). A water pump (Esotec GmbH, model “Fontana” with a flow rate maximum of 500 L/h) was mounted on the main unit and submerged to ensure a constant circulation of water around the immersed forearm. Finally, a custom made submergible, electronic button was placed inside the cooler to record the duration of time the participant stayed in the water (while a ceiling time of 3 min was imposed).

### Trier Social Stress Test

The TSST [17] is the most frequently administered psychological paradigm to stimulate an endocrine stress response in the laboratory setting. Compared to other social evaluative laboratory stressors, it provokes the most robust HPA-axis response [26]. It also increases hemodynamic responses [23], [27] and the activity of salivary alpha-amylase in a pattern resembling that of norepinephrine [28]. The TSST consists of three phases. A preparatory anticipation phase (5 min) is followed by a stress phase in which participants are asked to give a 5-min free speech for a simulated job interview followed by 5 min of difficult mental arithmetic. The entire performance is audio- and videotaped and held in front of an evaluative panel that does not provide any form of feedback. The TSST is usually preformed in a standing position. To provide a better comparability with the CPT, we here asked participants to sit down for the actual testing.

### Assessment and analysis of thermal IR images

IR-imaging was performed using a digital thermal camera (FLIR SC3000, FlirSystems, Sweden) with a Focal Plane Array of 320×240 QWIP detectors, 0.02 sec time resolution, 0.02 K temperature sensitivity and the capability to collect thermal radiation in the 8–9 μm band. In order to null noise effects related to the sensor drift/shift dynamics and optical artifacts, the camera response was blackbody-calibrated. The sampling rate was set at 5 frames/sec. Variations in cutaneous temperature of facial regions of interest were analyzed using customized Matlab programs (http://www.mathworks.com). Our primary regions of interest, 1) the nose, 2) the corrugator (supraorbital) and 3) the forehead, were selected based on previous studies in primates and humans, and have been shown to respond to emotional or distressing stimuli; responses in periorbital, perioral and chin regions were also analyzed [1], [2], [5], [29]–[31].

Initially, the thermal imprints were visually inspected to ensure adequate quality for all recordings. We then corrected the thermograms for body movements. In cases of marked head rotation, we skipped to the next available undistorted frame. Displacements between images were corrected frame by frame using anatomical landmarks based on the participants' nose profiles [32]. Visual inspection of the temperature time-course in the regions of interest evidenced short-lasting artifacts (potentially associated with transitions from nasal to oral breathing or vocalizations) which did not affect the long-term temperature evolution. Corrupted segments were filtered out following a procedure by Iriarte et al. [33]. Data were then processed with a band-stop filter to remove breathing-dependent oscillations. To provide evidence for a proper acclimatization of participants, we verified at the intra-individual level that the skin temperature did not vary significantly during the 5-min baseline phase.

### Assessment and analysis of heart rate, heart rate variability and finger temperature

Heart rate and HRV were derived from a continuous electrocardiogram (ECG) recorded with Biopac equipment (Biopac Systems, Inc.) using a three-electrode array. The peak of the R-wave was detected automatically to obtain a continuous RR-interval tachogram, which was corrected for artifacts manually when necessary. Heart rate was defined as the reciprocal of the RR-interval in units of beats per minute. Although heart rate combines sympathetic and parasympathetic contributions, adrenergic sympathetic influences are dominant during stress. Following guidelines put forth by the Task Force of the European Society for Cardiology [34], frequency domain analysis techniques were employed to investigate variations in the heart rate time series based on beat-to-beat intervals. The two main frequency bands of this signal are referred to as the low (0.04 to 0.15 Hz) and high frequency (0.15 to 0.4 Hz) components. High frequency components are believed to be influenced by parasympathetic tone while low frequency components are a combination of sympathetic and parasympathetic activities [35]. The ratio of low to high frequency components is used as a measure of sympathovagal balance [34], [35]. Finger temperature, an indicator of sympathetic activity [36], was sampled from the third finger of the right hand with a Biopac recording unit. All data were sampled at 500 Hz and analyzed with the software Acknowledge (version 3.9).

Heart rate (beats per min), HRV components (s^2^/Hz) and finger temperature change (slope) were extracted for each experimental phase (5 min for baseline, anticipation and recovery). For the TSST stress phase, interview and mental arithmetic were averaged to one 5-min stressor. For the CPT stress phase, a 5-min window including a maximum of 3 min of actual cold pain and a minimum of 2 min of recovery was used. Hence, the cardiovascular and thermal responses to the cold pain were likely underestimated.

### Assessment and analysis of salivary alpha-amylase

The salivary enzyme alpha-amylase which is mainly involved in the digestion of starch in the oral cavity has received increasing attention as an indicator of sympathetic activity within the past years [37]. Pharmacological studies have shown that its release is dependent on β-adrenergic transmission [38]. Using Salivette collection devices (Sarstedt, Nümbrecht, Germany), we sampled alpha-amylase at 10 min before and at 10 and 20 min after the onset of CPT and TSST to fully capture peak activity and return to baseline. Participants were instructed to place the saliva collection swabs in their mouths and to refrain from chewing for exactly 2 min [39]. Samples were stored at −30°C until analysis. Alpha-amylase activity (calculated and expressed in U/ml) was determined using an enzyme kinetic method [40],[41].

### Assessment and analysis of salivary cortisol

Cortisol is the most important endocrine stress marker. It was sampled using the same collection devices as for alpha-amylase. Samples were taken at 10 min before stressor onset, at 10 min after stressor onset and in 10-min intervals thereafter (until 70 min after stressor onset) to fully capture hormone peak and return to baseline. Cortisol concentration (calculated and expressed in nmol/l) was determined using a time-resolved fluorescence immunoassay [42], with intra- and interassay variabilities of less than 10% and 12%.

### Mood assessment

Stress-induced mood changes were assessed using two Visual Analogue Rating Scales (VARS) asking how anxious and angry participants currently felt on a scale ranging from 1 (not at all) to 10 (extremely). These VARS were administered at baseline (−10 min) and at the end of the anticipation phase/immediately before the onset of acute stress (0 min).

### Study design

**Figure 1:**
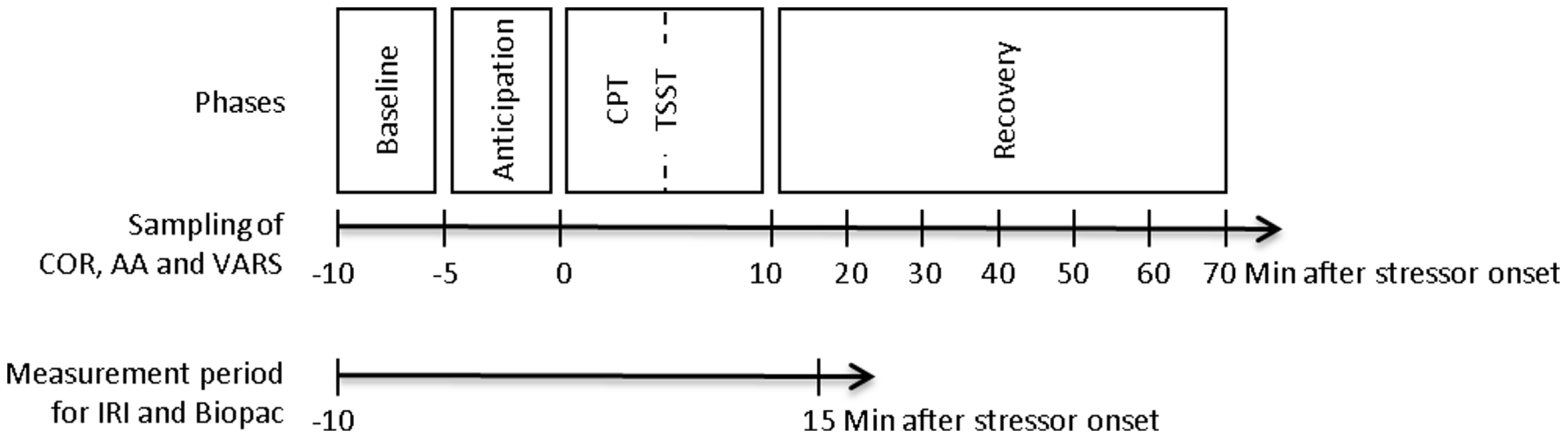
Timeline of the experiment. For the assessment of alpha-amylase (AA) and cortisol (COR), saliva was sampled at discrete measurement timepoints between −10 min and 20 min/70 min relative to stressor onset. Visual Analogue Rating Scales (VARS) assessing stress-induced mood changes were administered at baseline (−10 min) and at the end of the anticipation phase/immediately before the onset of acute stress (0 min). The measurement period for the thermal infrared imaging (IRI) and Biopac assessments began at −10 min and ended at 15 min relative to stressor onset.

Participants underwent CPT and TSST on two consecutive days in pseudo-randomized order. Testing took place at identical time slots on both days, either between 10:00 and 12:00 am or between 4:00 and 6:00 pm. Participants tested in the morning group were asked to get up at least 2 hours prior to coming to the laboratory. Figure 1 gives a detailed overview of the study design.

### Statistical analysis

#### Scoring of dependent variables

For the temperature measures, raw scores were defined as the mean slope per phase (baseline, anticipation, stress and recovery). For heart rate and HRV components, raw scores were defined as the arithmetic mean of the data within each experimental phase. To account for individual differences in baseline activity, reactivity and rebound scores were calculated as the change from baseline in each measure and phase. Alpha-amylase and cortisol data were initially Box-Cox-transformed to account for non-normal distribution and, in the case of the cortisol data, concentration-dependent variance due to HPA axis dynamics, immunoassay interference and measurement error [43]. Reactivity and rebound scores were calculated using the −10 min baseline, the mean peak (alpha-amylase: 10 min; cortisol: 30 min [CPT], 20 min [TSST]) and the final (alpha-amylase: 20 min; cortisol: 70 min) samples of the respective time series. Anticipatory increases are consequently missing for the alpha-amylase and cortisol data. For the mood ratings, change from baseline calculations used the baseline and the post-anticipation/pre-stress measures at −10 and 0 min. To conduct multivariate pattern analyses, the calculated difference scores were additionally z-standardized.

#### Univariate ANOVAs

We initially examined which thermal imprints and established stress markers were sensitive for change over time in CPT and TSST. One-way repeated measures ANOVAs were calculated for the dependent variables (raw or logged scores) in each stress test. As within-subject factors, sampling time-points (−10 to 20 and 70 min, respectively) were entered for alpha-amylase and cortisol, and phases (baseline, anticipation, stress and recovery) for all other dependent variables. Violations of the assumption of sphericity were adjusted using the Huynh-Feldt correction. Critical p-values were corrected for multiple comparisons using the Bonferroni adjustment (α/n) per stress test. Partial eta-squared (η_p_^2^) was used as an effect size estimate. Significant effects were further investigated using simple contrasts with the baseline as reference category.

#### Correlation analyses

To examine whether facial thermal imprints and established stress markers captured similar aspects of the stress response, Pearson correlations between the change-sensitive candidates as found in the univariate ANOVAs were calculated for each stress test and phase. As indicators of psychophysiological covariance, correlations between the change-sensitive candidates and anticipatory mood changes were calculated per stress test and test phase. Critical p-values were again corrected for multiple comparisons using the Bonferroni adjustment (α/n).

#### Multivariate pattern analyses

The next set of analyses determined the predictive power of our two sets of dependent variables for the three phases of the stress cycle. In detail, we examined how well the composite multivariate response patterns of either the thermal imprints or the established stress markers characterized, and in consequence predicted, a specific test phase. For this purpose we used an advanced multivariate pattern analysis approach that was developed in machine learning. MVPAs are considered particularly sensitive to the detection of differential response patterns across multiple variables, a quality which allows them to reliably characterize complex physiological and mental states. So far, they have been applied successfully in diverse research areas ranging from neuroscience to chemistry [44]–[47].

MVPAs were applied separately for CPT and TSST. Four sets of analyses were run. The first analysis tested whether the response patterns of the established stress markers collected throughout anticipation, stress and recovery phases of the CPT allowed predicting which phase of the stress cycle a participant was currently experiencing (MVPA 1). The same analysis was then performed using the response patterns of the thermal imprints collected throughout all CPT phases (MVPA 2). In a parallel manner, MVPAs 3 and 4 examined whether established stress markers or thermal imprints formed characteristic responses profiles to the phases of the TSST. Since anticipation scores for alpha-amylase and cortisol were not collected, MVPAs 1 and 3 were based on only five established stress markers.

**Figure 2:**
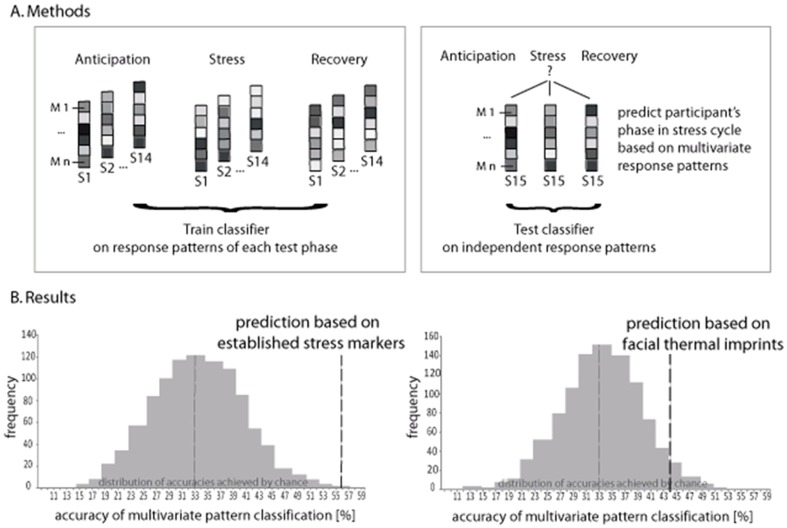
Multivariate pattern analysis of phases in the stress cycle. A Methods. For each subject (S_1…15_), responses patterns were created that included the n measures (M) of either established stress markers or thermal imprints of a particular test phase (anticipation, stress, recovery). Response patterns of all but one subject were used to train a classifier to identify physiological response profiles that are characteristic for each of the test phases. This was the basis for the subsequent prediction of the response patterns of the remaining subject as belonging to either anticipation, stress of recovery phase. B Results. Multivariate response patterns of established stress measures obtained for the CPT (MVPA 1, left) reliably predicted the current test phase of a subject well above chance (56% classification accuracy, p<.001). Patterns of thermal facial imprints in the CPT (MVPA 2, right), on the other hand, did not significantly predict the correct phase (40% classification accuracy, p = .13). The histograms display the permutation distribution of classification accuracies achieved by chance (average 33%).

For each MVPA, three separate N-dimensional pattern vectors were created that contained the N (IR-imaging or established) variables per participant obtained throughout the three test phases (Figure 2). Using a conservative cross-validation procedure, the resulting pattern vectors of all except one participant were used to train a linear support vector machine classifier (i.e., estimate a decision boundary that separates phase-specific response patterns) with a fixed regularization parameter C = 1 (“training data”). The classification accuracy of the response patterns as belonging to either the anticipation, stress or recovery phase was determined depending on which phase the respective response patterns actually did belong to. This procedure was repeated 15 times, always omitting the pattern vectors of another participant from the training. The average percentage of correct classification across the resulting 15-fold cross-validation steps represented the predictive strength of the multivariate response patterns for the three test phases. Using a Wilcoxon test, the average classification accuracy of each analysis (MVPA 1–4) was then statistically tested against a respective permutation distribution of classification accuracies. In so doing, we determined the probability with which the average classification accuracy was achieved by chance only. Given three options (phases) to choose from, chance level was at 33%. Using 1000 repetitions, each permutation distribution was obtained by randomly assigning the “training data” to one of the three test phases. The MVPAs were performed using MATLAB R2012b and the libSVM toolbox (http://www.csie.ntu.edu.tw/~cjlin/libsvm/). All remaining tests were performed using the Predictive Analysis Software (PASW) version 17.

## Results

### Univariate ANOVAs

**Figure 3:**
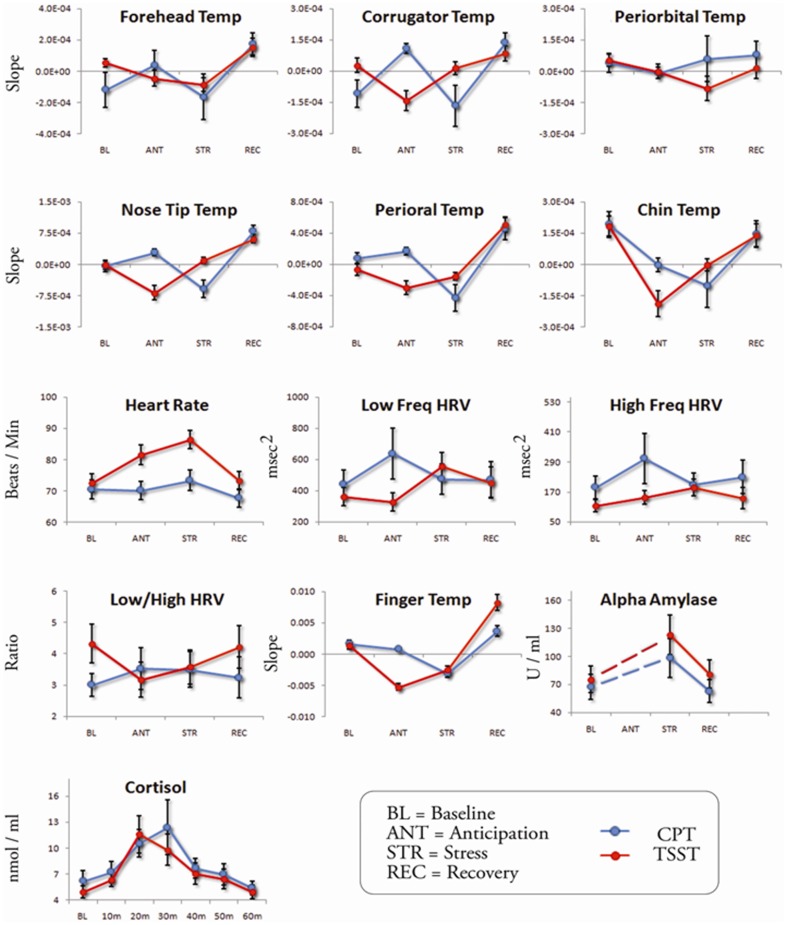
Means and ±SEM of all dependent variables (raw or logged scores) during baseline, anticipation, stress and recovery (sampling time points for alpha-amylase and cortisol) of CPT and TSST.

For each dependent variable, change sensitivity in CPT and TSST was examined in altogether 13 ANOVAs per stress test (critical p-values were lowered to .004). Both thermal imprints and established stress markers were sensitive to depict change in CPT and TSST (see Table 2 and Figure 3 for the summary statistics and time courses of all variables). In the CPT, nose tip temperature, perioral temperature, heart rate and finger temperature showed significant changes over time. Posthoc tests revealed that none of the variables changed from baseline to anticipation. Relative to the baseline, nose tip, perioral and finger temperatures decreased during stress and nose tip temperature increased during recovery. Inversely, heart rate increased during stress and decreased during recovery. In the TSST, corrugator temperature, nose tip temperature, perioral temperature, chin temperature, heart rate, finger temperature, alpha-amylase and cortisol showed significant changes over time. Posthoc tests revealed that relative to the baseline, corrugator, nose tip, chin and finger temperatures decreased during anticipation. Chin and finger temperatures remained significantly decreased during stress. Nose tip, perioral and finger temperatures increased during recovery. Heart rate increased during anticipation and increased further during stress. Alpha-amylase levels increased from baseline to 10 min after stressor onset. Cortisol levels increased until they reached a peak at 20 min after stressor onset and decreased again thereafter.

**Table 2:** Univariate statistics (one-way repeated measures ANOVAS with simple contrasts) for raw (logged for alpha-amylase and cortisol) scores over time in CPT and TSST.

	Variable	F (*df*), p^a^, η_p_^2^	F(*df*), P for post-hoc tests (simple contrasts)^b^		
			BL-ANT	BL-STR	BL-REC
**CPT**					
	**Facial thermal imprints**				
	FHT	1.60 (3, 42), >.10, .10			
	CRT	4.57 (3, 42), .009, .23			
	POBT	0.25 (3, 42), >.30, .02			
	NTT	***13.31 (3, 42), <.001, .47***		6.23 (1, 14), p = .025 (↓)	14.54 (1, 14), p = .002 (↑)
	POT	***7.48 (3, 42), .004, .33***		9.61 (1, 14), p = .007 (↓)	
	CHT	3.11 (3, 42), .033, .17			
	**Established stress markers**				
	HR	***8.28 (3, 42), .001, .37***		6.71 (1, 14), p = .021 (↑)	6.99 (1, 14), p = .019 (↓)
	LF	1.75 (3, 42), >.10, .11			
	HF	1.73 (3, 42), .094, .11			
	LF/HF	0.44 (3, 42), .30, .03			
	FT	***19.18 (3, 42), <.001, .56***		25.16 (1, 14), p<.001 (↓)	
	AA	4.03 (2, 28), .014, .21			
	COR	***7.24 (6, 84), .001, .33***		BL-20 min: 12.94 (1, 14), p = .003 (↑)	
				BL-30 min: 7.49 (1, 14), p = .015 (↑)	
**TSST**					
	**Facial thermal imprints**				
	FHT	4.10 (3, 42), .012, .21			
	CRT	***5.49 (3, 42), .003, .27***	7.43 (1, 14), p = .016 (↓)		
	POBT	1.47 (3, 42), >.10, .09			
	NTT	***16.42 (3, 42), <.001, .52***	8.52 (1, 14), p = .011 (↓)		16.58 (1, 14), p = .001 (↑)
	POT	***15.76 (3, 42), <.001, .51***			14.74 (1, 14), p = .002 (↑)
	CHT	***9.24 (3, 42), <.001, .38***	13.52 (1, 14), p = .002 (↓)	24.63 (1, 14), p<.001 (↓)	
	**Established stress markers**				
	HR	***29.11 (3, 42), <.001, .68***	22.92 (1, 14), p<.001 (↑)	55.35 (1, 14), p<.001 (↑)	
	LF	3.60 (3, 42),.011, .22			
	HF	2.28 (3, 42),.067, .15			
	LF/HF	2.10 (3, 42),.058, .14			
	FT	***46.94 (3, 42), <.001, .76***	44.09 (1, 14), p<.001 (↓)	28.71 (1, 14), p<.001 (↓)	38.51 (1, 14), p<.001 (↑)
	AA	***7.61 (2, 28), .001, .34***		BL-10 min: 17.79 (1, 14), p<.001 (↑)	
	COR	***17.79 (6, 84), <.001, .54***		BL-10 min: 11.93 (1, 14), p = .004 (↑)	
				BL-20 min: 24.23 (1, 14), p<.001 (↑)	
				BL-30 min: 13.93 (1, 14), p = .002 (↑)	
				BL-40 min: 4.86 (1, 14), p = .044 (↑)	

*Note*. AA: alpha-amylase; ANT: anticipation; BL: baseline; CHT: chin temperature; COR: cortisol; CRT: corrugator temperature; FHT: forehead temperature; FT: finger temperature; HF: high frequency heart rate variability; HR: heart rate; LF: low frequency heart rate variability; LF/HF: ratio of low to high frequency heart rate variability; NTT: nose tip temperature; POBT: periorbital temperature; POT: perioral temperature; REC: recovery; STR: stress.

a after multiple comparisons correction p≤.004.

b ↑: ANT, STR, REC > BL; ↓: ANT, STR, REC < BL.

### Correlation analyses

No significant correlations between thermal imprints and established stress markers were found in anticipation, stress and recovery phases of either the CPT or the TSST (Table 3; based on the maximum number of change-sensitive measures per stress test and test phase, critical p-values were lowered to ≤.005 for the anticipation phase of the TSST, the stress phases of both tests and to p≤.050 and ≤.017 for the recovery phases of CPT and TSST in order to control for multiple comparisons). Even within each group of measures, significant associations were scarce. In detail, in the CPT, relative-to-baseline changes in nose tip and perioral temperatures during acute stress correlated at r = .60 (p = .007). In the TSST, relative-to-baseline changes in corrugator and chin temperatures during anticipation correlated at r = .62 (p = .005), changes in heart rate and cortisol during acute stress correlated at r = .73 (p = .001) and changes in nose tip and perioral temperatures during recovery correlated at r = .82 (p<.001).

**Table 3:** Tables 3. Pearson correlations between the change-sensitive candidates for each test phase in CPT and TSST.

	Variable	r, p^a^				
**CPT**						
	**Stress**					
	NTT	NTT	POT	HR	FT	COR
	POT	1	**.** ***60, .007***	−.26, .172	.34, .098	−.41, .058
	HR		1	.30,.140	.10, .357	.47, .032
	FT			1	.13, .327	.20, .236
	COR				1	.02, .478
	NTT					1
	**Recovery**					
		NTT	HR			
	NTT	1	−.13, .320			
	HR		1			
**TSST**						
	**Anticipation**					
		NTT	CRT	CHT	HR	FT
	NTT	1	.02, .469	.14, .198	.02, .473	.49, .028
	CRT		1	**.** ***62, .005***	.28, .160	.11, .227
	CHT			1	.22, .216	.03, .460
	HR					−.19, .236
	FT					1
	**Stress**					
		CHT	HR	FT	AA	COR
	CHT	1	.30, .141	−.17, .263	−.39, .068	−.11, 349
	HR		1	−.01, .484	.29, .137	**.** ***73, .001***
	FT			1	.17, .262	.01, .496
	AA				1	.37, .072
	COR					1
	**Recovery**					
		NTT	POT	FT		
	NTT	1	**.** ***82, <.001***	.22, .209		
	POT		1	−.12, .335		
	FT			1		

*Note*. AA: alpha-amylase; CHT: chin temperature; COR: cortisol; CRT: corrugator temperature; FT: finger temperature; HR: heart rate; NTT: nose tip temperature; POT: perioral temperature.

a after multiple comparisons correction, p≤.005 for the anticipation phase of the TSST, the stress phases of both tests and p≤.050/.017 for the CPT/TSST recovery phases.

Psychophysiological covariance (i.e. covariance of stress-induced psychological and physiological responses) emerged only for the thermal imprints and only in the TSST (Table 4; to correct for multiple comparisons, critical p-values were lowered to .010 for the anticipation phase of the TSST, the stress phases of both CPT and TSST, and to .025 and .017 for the CPT and TSST recovery phases). Anxiety was inversely correlated with anticipation-induced (r = −.65, p = .003) and acute stress-induced (r = −.60, p = .007) changes in chin temperature. Anger correlated with anticipation-induced changes in corrugator (r = −.69, p = .002) and chin (r = −.72, p = .001) temperatures.

**Table 4:** Pearson correlations between the change-sensitive candidates and the anticipatory mood changes (anxiety and anger) per test phase in CPT and TSST.

	Variable	r, p^a^	
		Anxiety	Anger
**CPT**			
	**Stress**		
	NTT	.45, .042	.54, .015
	POT	.23, .194	.28, .151
	HR	−.36, .097	−.34, .110
	FT	−.45, .042	.24, .185
	COR	.05, .428	−.01, .491
	**Recovery**		
	NTT	.13, .312	.17, .260
	HR	.42, .060	−.20, .243
**TSST**			
	**Anticipation**		
	NTT	−.16, .280	.00, .500
	CRT	−.40, .065	**−.** ***69, .002***
	CHT	**−.** ***65, .003***	**−.** ***72, .001***
	HR	−.13, .315	−.32, .114
	FT	.15, .277	−.03, .449
	**Stress**		
	CHT	**−.** ***60, .007***	−.51, .022
	HR	−.32, .115	−.46, .037
	FT	.11, .340	.17, .262
	AA	.30, .122	.14, .297
	COR	−.03, .455	−.04, .441
	**Recovery**		
	NTT	−.05, .430	−.02, .466
	POT	−.12, .323	−.14, .304
	FT	−.03, .461	.04, .447

*Note*. AA: alpha-amylase; CHT: chin temperature; COR: cortisol; CRT: corrugator temperature; FT: finger temperature; HR: heart rate; NTT: nose tip temperature; POT: perioral temperature.

a after multiple comparisons correction, p≤.010 for the anticipation phase of the TSST, the stress phases of both tests and p≤.025/.017 for the CPT/TSST recovery phases.

### Multivariate pattern analyses

Using multivariate pattern analyses (MVPAs), we investigated the capacities of thermal imprints and established stress markers to discriminate the phases of the stress cycle and reliably predict whether a participant was currently anticipating a stressor, exposed to a stressor or in the process of recovering from it. In the CPT, patterns of established stress markers were found to reliably characterize phase-specific stress responses and thus predict test phases well above the chance level of 33% (56% average classification accuracy across participants, p<.001; MVPA 1) (Figure 2). In other words, the established stress markers formed unique response profiles for anticipation, stress and recovery phases that could be used to correctly predict which phase of the stress cycle a person was currently exposed to. Patterns of thermal imprints in the CPT, on the other hand, did not allow discriminating and correctly predicting a corresponding test phase (MVPA 2): The average classification accuracy of 40% was not significantly different from predictions achieved by chance only (p = .13) (Figure 2). This means that the thermal imprints formed fairly similar response profiles for anticipation, stress and recovery phases. Parallel results were obtained for the TSST. While the response patterns of established stress markers allowed to reliably predict the correct test phase well above chance (56%, p<.001; MVPA 3), this was not possible for the response patterns of thermal imprints (38%, p>0.40; MVPA 4). Taken together, there was not enough specificity in the response patterns of the thermal imprints to give each phase its discriminable profile.

## Discussion

The goal of this pilot study was to test the use of thermal IR-imaging in human stress research. Hereby, we compared how facial thermal imprints and established stress markers performed in two frequently applied laboratory stress tests, the CPT and the TSST.

Univariate ANOVAs revealed that indeed most thermal imprints changed significantly from baseline in the course of CPT and/or TSST. More specifically, out of six facial thermal imprints, only forehead and periorbital temperatures turned out to be overall stress-insensitive. Among the remaining markers (corrugator, nose tip, perioral and chin temperatures) the measure most frequently yielding significant results in both stress tests was nose tip temperature. Regarding the superior change sensitivity of the nasal region, our findings are in line with previous studies in which infants [31], children [5] and adults [29] were confronted with emotionally arousing stimuli. However, the observed insensitivity of the forehead and periorbital regions does not concur with the literature [2], [29] and may be specific to the present context in stress research. Overall, the facial thermal imprints yielded a similar range of small to medium effect sizes than the established stress markers.

Correlation analyses revealed that there were no significant associations between the change-sensitive thermal imprints and established stress markers in either of the tests phases or stress tests. Importantly, this does not automatically suggest that the thermal imprints provide an invalid measure of stress. For one thing, an N of 15 is at the lower end for computing correlations. However, the lack of association between stress markers is also a well-known issue in stress research and has been attributed to the fact that different markers originate from different stress-related systems and underlie different time courses [21], [23], [48]. Accordingly, also within each group of measures, significant correlations were scarce. We conclude that the thermal imprints and established stress markers capture unique aspects of physical and psychological stress responses.

Correlation analyses additionally showed overall weak psychophysiological covariance (i.e. covariance of stress-induced psychological and physiological responses). Only corrugator and chin temperatures but none of the established stress markers correlated with stress-induced mood changes. Again, this result may, at least partially, be due to our relatively small number of participants but simultaneously reflects a phenomenon known in the literature [48]–[50] and has been ascribed to both the time lag between psychological and physiological stress responses and general difficulties in the assessment of subjective stress responses by self-report methods [50], [51]. In this regard, it is especially interesting that the facial thermal imprints (precisely, corrugator and chin temperatures) did capture the experiential nature of stress. Possibly, because of evolving at the very surface of our body, facial temperature variations are more easily discerned than other, internally evolving, physiological processes and consequently exert an important influence on our judgments of stress-induced mood changes.

MVPAs revealed that in contrast to the established autonomic stress markers, the thermal imprints were not able to reliably discriminate between anticipation, stress and recovery phases of CPT and TSST. In other words, there was not enough specificity in their multivariate response patterns to give each phase its discriminable profile. We speculate that the thermal imprints reflect the general, unspecific arousal that underlies the entire stress experience and is hence common to all phases of the stress cycle. The established autonomic stress markers, on the other hand, seem to form a more heterogeneous group as they pick up on the specific physiological patterns associated with only anticipation, stress or recovery phases in both stress tests. Importantly, cortisol as the hallmark endocrine biomarker of stress was not included in this set of analysis and therefore does not contribute to the heterogeneity of the group of established stress markers.

Several methodological limitations have to be addressed. One critical point concerns the small sample size of N = 15. Until replicated in a bigger sample, data have to be considered preliminary. Another critical point is the lack of a control condition to depict the course of normal fluctuations in facial thermal activity over time. However, two facts speak against the possibility that we measured normal variations rather than stress-induced change. For one thing, we found response cycles in most of our dependent variables, i.e. participants started from a baseline, responded to the experimental manipulation and then returned back to their baseline. Additionally, finding correlations between thermal imprints and stress-induced psychological responses would have been unlikely in the context of normal fluctuations in thermal activity. The fact that exclusion criteria were determined by telephone interview is another limitation. Ideally, participants should have been screened for psychological issues and somatic conditions by use of a detailed clinical diagnostic interview and a medical examination, respectively. Lastly, the fact that we used two different time windows for testing – one in the late morning and one in the afternoon – is not ideal given that especially cortisol follows a strong circadian rhythm [52], [53]. Although it was assured for all participants that testing took place at identical time slots on both days, non-significant results may nevertheless be due to differences in baseline values and excitability of the examined physiological systems in the morning versus afternoon hours.

In summary, this study makes several important contributions to the literature. We provide a first-time verification of the usefulness of thermal IR-imaging in the classical stress laboratory setting. Thermal imprints (precisely, corrugator, nose tip, perioral and chin temperatures) reliably depict stress-induced change over time. While nose tip and perioral temperatures are the strongest candidates when it comes to predicting change, correlation analyses show that corrugator and chin temperatures also capture the experiential nature of anticipatory and acute stress. In contrast to the established stress markers, the thermal imprints form a non-specific set of measures that respond in a similar way to the different phases of the stress cycle. We conclude that when looking to characterize complex physiological states over the time course of a given stress cycle, the established autonomous stress markers (e.g., heart rate and finger temperature) are more suitable than the thermal imprints. If, however, research aims for covert recordings or focusses on special populations which show difficulties in complying with the standard instruments of data collection, contact-free, non-invasive thermal IR-imaging may be the method of choice. The use of IR-imaging may also be particularly beneficial in the domain of psychophysiological covariance research. Modern thermal imaging systems can provide adequate temperature sensitivity and spatial resolution without being dramatically expensive (the cost of an average performance system does not exceed the price of good-quality biosignal recording systems). Also, the employment of thermal IR-imaging does not require any time-consuming preparations or special skills. The field would profit from the development of software packages facilitating data analysis, however. Overall, this technique could be easily integrated in neuro-psycho-physiology experimental settings.

Our analyses additionally give an overview of the potency with which CPT and TSST trigger responses in the different phases of the stress cycle, and how a large range of stress markers respond per stress test and phase. This overview may serve as a useful tool for the targeted selection of the most potent combinations of tests and markers in future research. Both the ANOVA and the correlation results, for example, illustrate that a psychosocial challenge is more qualified than a physical challenge to bring out anticipatory stress. In the study of anticipatory stress, focusing on the TSST and assessing heart rate and temperature indices would thus be indicated. Finally, we present a new multivariate analysis approach in behavioral stress research. The claim has been raised that to accurately represent the multidimensional nature of stress, multiple measures across multiple branches of the stress system should be assessed [54]. MVPAs provide an elegant method for exploring complex data sets: Instead of considering the correlations between single variables, they make predictions based on the underlying patterns across numerous variables. In the field of stress research, the application of MPVAs to predict vulnerability for psychopathology based on various indices of stress sensitivity, for example, would constitute an exiting future usage of this technique.
